# Is adjunctive pharmacotherapy in attention-deficit/hyperactivity disorder cost-effective in Canada: a cost-effectiveness assessment of guanfacine extended-release as an adjunctive therapy to a long-acting stimulant for the treatment of ADHD

**DOI:** 10.1186/s12888-016-0708-x

**Published:** 2016-01-16

**Authors:** Jean Lachaine, Vanja Sikirica, Karine Mathurin

**Affiliations:** Faculty of Pharmacy, University of Montreal, P.O. Box 6128, Station Centre-ville, Montreal, Quebec H3C 3J7 Canada; Shire, 725 Chesterbrook Boulevard, Wayne, PA 19087 USA

**Keywords:** Attention-deficit/hyperactivity disorder, Guanfacine extended-release, Stimulants, Adjunctive therapy, Pediatrics, Canada, Cost-effectiveness, Cost-utility

## Abstract

**Background:**

Attention-deficit/hyperactivity disorder (ADHD) is a common psychiatric disorder in children, with worldwide prevalence of ADHD varying from 5.9 to 7.1 %, depending on the reporter. In case of inadequate response to stimulants, combination therapy of stimulants and an adjunctive medication may improve the control of ADHD symptoms, reduce the dose-limiting adverse events, and help control comorbidities. To date, the only medication to be used for adjunctive therapy to psychostimulants is guanfacine extended release (GXR). The aim of this study was to assess the economic impact of GXR as an adjunct therapy with long-acting stimulants (GXR + stimulant) compared to long-acting stimulant monotherapy (stimulant alone) in the treatment of children and adolescents with ADHD in Canada.

**Method:**

A Markov model was developed using health states defined based on the clinician-reported Clinical Global Impression-Severity (CGI-S) score (normal, mild, moderate, severe). Transition probabilities were calculated based on patient-level data from a published study. Long-acting stimulants available in Canada were considered in the base-case model: amphetamine mixed salts, methylphenidate HCl formulations, and lisdexamfetamine dimesylate. Analyses were conducted from a Canadian Ministry of Health (MoH; Ontario) and a societal perspective over a 1-year time horizon with weekly cycles.

**Results:**

Over a 1-year time horizon, GXR + stimulant was associated with 0.655 quality-adjusted life year (QALY), compared to 0.627 QALY with stimulant alone, for a gain of 0.028 QALY. From a MoH perspective, GXR+ stimulant and stimulant alone were associated with total costs of $CA1,617 and $CA949, respectively (difference of $CA668), which resulted in an incremental cost-effectiveness ratio (ICER) of $CA23,720/QALY. From a societal perspective, GXR + stimulant and stimulant alone were associated with total costs of $CA3,915 and $CA3,582, respectively (difference of $CA334), which resulted in an ICER of $CA11,845/QALY. Probabilistic sensitivity analysis (PSA) of GXR + stimulant showed that it remains a cost-effective strategy in 100 % of the simulations from both perspectives in numerous PSA and one-way sensitivity analyses, relative to a willingness to pay threshold of $50,000/QALY.

**Conclusions:**

This economic evaluation demonstrates that GXR + stimulant is cost-effective compared to stimulant alone in the treatment of children and adolescents with ADHD in Canada.

## Background

Attention-deficit hyperactivity disorder (ADHD) is among the most common psychiatric disorder in children, with an overall Canadian prevalence in children that has increased from 1.3 % in 1994–1995 to 2.1 % in 2008–2009 [1]. The worldwide prevalence of ADHD varying from 5.9 to 7.1 %, depending on the reporter [2]. As ADHD is associated with a substantial clinical burden, it imposes a significant economic impact on the health care system and society. In fact, ADHD is associated with an increased risk of substance use, a lower academic performance and occupational status, absenteeism, and productivity loss [3–5]. According to the Centre for ADHD Awareness, Canada, the total economic burden associated with ADHD in children would reach $CA2 billion per year based on US cost estimates [6]. More recently, a systematic review by Doshi et al. indicated that the overall annual incremental costs of ADHD in the US ranged from $US143 to $US266 billion, of which between $US38 and $US72 billion were incurred by children and adolescents [7].

The availability of effective ADHD treatments may contribute to reduce the substantial psychosocial and economic burden of the disorder. According to recent Canadian guidelines, long-acting stimulants are recommended as the first-line treatment of ADHD in children aged from 6 to 12 years [8]. However, approximately 30 % of children with ADHD do not have an adequate response to a single stimulant, often defined as a percentage improvement on the ADHD Rating Scale IV (ADHD-RS-IV) or change in Clinical Global Impression-Improvement score [9, 10]. However, for many reasons, such as having an inadequate or partial response and dose-limiting side effects, some patients with ADHD augment their existing stimulant with additional medications [11, 12]. A recent Canadian study found that children and adolescents with ADHD treated with stimulants in Quebec had rates of adjunctive therapy of 19.8 and 18.7 %, respectively [13]. A combination therapy comprising multiple stimulants (treatments initially given together) and an adjunctive medication (a second treatment added to the initial treatment) may improve the control of core ADHD symptoms, reduce the dose-limiting adverse events (AEs) associated with stimulants, and help control comorbidities (including mood, anxiety, and substance disorders) [9]. In a Canadian retrospective claims analysis, approximately one in five children/adolescents with ADHD were on a stimulant experienced combination therapy [14]. Despite the relatively high prevalence of adjunctive therapy among stimulant users, there are limited clinical trial data to support the use of a stimulant and another medication as adjunctive therapy.

To date, in Canada, the only medication approved by Health Canada for adjunctive therapy to stimulants in children aged 6–12 years is Intuniv XR™ (guanfacine extended release [GXR]; Shire Canada Inc., Saint-Laurent, QC, Canada). The efficacy and tolerability of GXR as an adjuctive therapy has been demonstrated in randomized placebo controlled clinical trials [15, 16]. In these studies, GXR treatment groups showed significantly greater symptom reduction from baseline as measured by the ADHD-RS-IV total score compared to placebo plus stimulant at endpoint.

GXR as an adjunctive therapy to existing simulant monotherapy has been shown to be cost-effective compared to existing stimulant monotherapy alone in a US context [17]. However, the cost-effectiveness of GXR as an adjunctive therapy for the treatment of ADHD has not been evaluated in Canada. Therefore, the aim of this study was to perform a cost-effectiveness analysis (CEA) comparing GXR as an adjunctive therapy to stimulants with stimulant monotherapy among children with ADHD who had a suboptimal response to stimulants in a Canadian economic context.

## Methods

A CEA was performed to assess the economic impact of GXR as an adjunctive therapy to long-acting stimulants in the treatment of ADHD. This economic evaluation was based on the recently published results from a randomized study by Wilens et al., which compared GXR as an adjunctive therapy to stimulants with placebo plus stimulants (no distinction between stimulants) [16]. It was assumed that the target population, treatment efficacy, and tolerability profile used in the US clinical trial was generalizable to a similar ADHD target population in Canada.

### Comparative treatment

According to Canadian ADHD treatment guidelines, long-acting stimulants are the mainstays of pharmacological therapy and are first-line treatment options for the majority of patients with ADHD [8]. Specifically, Adderall XR^®^ (amphetamine mixed salts [MAS-XR]; Shire Canada Inc.), Concerta^®^ (methylphenidate HCl extended release [OROS-MPH]; Janssen Inc., Toronto, ON, Canada) and generic, Biphentin^®^ (methylphenidate HCl controlled release [MPH-CR]; Purdue Pharma, Pickering, ON, Canada) and Vyvanse^®^ (lisdexamfetamine dimesylate [LDX]; Shire Canada Inc.) are available in Canada for the treatment of ADHD [18, 19]. In case of suboptimal response to long-acting stimulants, an adjunctive medication can be added. Therefore, in the base-case analysis of this CEA, GXR as an adjunctive therapy to a long-acting stimulant was compared to long-acting stimulant monotherapy.

In the pivotal US study, GXR or placebo was co-administered to a long-acting stimulant in patients who had a suboptimal response to stimulants alone [16]. Suboptimal response was defined as follows: **≥**4 weeks of a stable dose of treatment with an extended-release stimulant with improvement but continued mild to moderate symptoms of ADHD; ADHD-RS-IV total score of **≥**24 and a Clinical Global Impression-Severity (CGI-S) score indicative of at least mild impairment (**≥**3); and investigator assessment of inadequate response to current stimulant. To represent the Canadian situation, only long-acting stimulants available in Canada were considered in the base-case model: MAS-XR, OROS-MPH, MPH-CR and LDX [8]. As Biphentin^®^, a methylphenidate-based stimulant, is not available in the US and was not assessed in the study by Wilen et al., a similar efficacy for Biphentin^®^ and other methylphenidates included in the pivotal study was assumed, as supported by literature [20].

### Target population

The study population in the economic evaluation consisted of children with ADHD aged 6–12 years with a suboptimal response to stimulants, according to the product label indication in Canada for GXR. However, in the study by Wilens et al., the target population was children and adolescents aged 6–17 years [16]. Within the Wilens et al. study, changes in ADHD-RS-IV mean total score from baseline to endpoint were similar between age groups (6–12 and 13–17 years old) and no efficacy data were stratified by the child versus adolescent age groups. In the model, it was assumed that the clinical efficacy of GXR was similar to that measured in Wilens et al. The mean age of the trial population was 10.8 years (79.3 % aged 6–12 years; 20.7 % aged 13–17 years) and the proportion of male patients was 71.6 %.

### Model structure

**Fig. 1:**
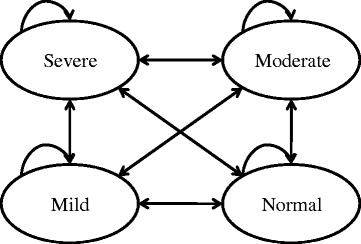
Diagram of health states. Patients may enter the model in the mild, moderate, or severe states. Adapted from Sikirika et al. 2012

A two-stage Markov model was developed over a 1-year time horizon with weekly cycles, which is in line with other CEAs of ADHD treatments [21–24]. Markov health states were defined based on the clinician reported CGI-S scores and included the following stages of the disease: severe (CGI-S score of “Severely ill” or “Among the most extremely ill subjects”), moderate (CGI-S score of “Moderately ill” or “Markedly ill”), mild (CGI-S score of “Borderline ill” or “Mildly ill”) and normal (CGI-S score of “Normal”) (Fig. 1). Patients’ starting health state was based on the distribution of starting CGI-S scores in the trial across the treatment arms (Table 1).

**Table 1:** Key Model Inputs

Parameter	Value	Source
Initial health distribution:		
Normal	0.00 %	Phase III trial [16]
Mild	3.52 %	
Moderate	90.55 %	
Severe	5.93 %	
Utility inputs:		
Normal	0.839	Lloyd et al. [31]
Mild	0.787	
Moderate	0.578	
Severe	0.444	
Weekly medical costs:		
Normal	$4.71	Derived from Guevara et al. [25], Schedule of Benefit and Fees, OHIP, OCCI
Mild	$4.76	
Moderate	$13.63	
Severe	$28.38	
Weekly costs associated with productivity losses for parents of children with ADHD (societal perspective):		
Normal	$14.60	Hakkaart-van Roijen et al. [29] Statistics Canada
Mild	$15.38	
Moderate	$60.30	
Severe	$125.10	
Daily cost of ADHD medication, $CA:		
GXR	$3.89	Quebec’s Medication List
MAS-XR (Adderall XR^®^)	$3.24	
MPH-CR (Biphentin^®^)	$1.77	ODB
OROS-MPH (Concerta^®^)	$2.76	Market shares – Canada
OROS-MPH (generic)	$1.91	(except Quebec), IMS Brogan
LDX (Vyvanse^®^)	$3.75	
Long-acting stimulants - Overall	$2.80	Weighted average cost
Percentage of patients taking long-acting stimulants:		
GXR	–	Market shares – Canada (except Quebec), IMS Brogan
MAS-XR (Adderall XR^®^)	14.20 %	
MPH-CR (Biphentin^®^)	15.30 %	
OROS-MPH (Concerta^®^)	46.10 %	
OROS-MPH (generic)	6.40 %	
LDX (Vyvanse^®^)	18.00 %	

*ADHD* attention-deficit/hyperactivity disorder, *GXR* guanfacine extended release, *LDX* lisdexamfetamine dimesylate, *MAS-XR* mixed amphetamine salts extended release, *MPH-CR* methylphenidate hydrochloride controlled release, *OCCI* Ontatio Case Costing Initiative, *ODB* Ontario Drug Benefit Formulary, *OHIP* Ontario Health Insurance Plan, *OROS-MPH* osmotic release oral system methylphenidate

Consistent with the trial period, the first stage of the model was assumed to span from week 0 to week 8, and the second stage extended from week 9 to week 52. All patients remained on treatment during the first stage of the model. Thereafter, patients in the moderate or severe states at week 8 were considered to be non-responsive and therefore permanently discontinued their treatments. As most of the patients included in the trial had moderate or severe disease at baseline, remaining with a moderate or severe disease after 8 weeks would indicate a lack of response to treatment. Similarly, patients who transitioned into the moderate or severe state during the second stage of the simulation (weeks 9–52) discontinued treatment and remained in the last observed health state for the rest of the model period. In a sensitivity analysis, patients were maintained on treatment and could transition between heath states during the weeks 9–52 period.

### Transition probabilities

The transition probabilities between health states were taken from the patient-level data from the pivotal study, as previously described [7]. Briefly, following the trial definition of endpoint, the efficacy data from the first 8 weeks were used. Patients were assigned each week to one of the four health states from week 0 to week 8 based on the observed weekly CGI-S values.

In the base-case model, ordered logit models were used to estimate the transition probabilities, where the dependent variable was the current health state and the independent variable was the health state in the previous week (Table 2). Transition probabilities were estimated for the placebo plus psychostimulants arm and the combined GXR plus psychostimulants arm for morning and evening administration. The estimated transition probabilities were applied throughout the model period for patients remaining on treatment (e.g. 9–52 weeks).

**Table 2:** Transition probabilities based on logit model

CGI-S health states	CGI-S health states in Subsequent Week			
	Normal	Mild	Moderate	Severe
Stimulants only (mean SE)				
Normal	0.759 (0.056)	0.238 (0.055)	0.003 (0.001)	0.000 (0.000)
Mild	0.081 (0.013)	0.826 (0.017)	0.093 (0.014)	0.000 (0.000)
Moderate	0.002 (0.001)	0.192 (0.015)	0.801 (0.015)	0.004 (0.003)
Severe	0.000 (0.000)	0.000 (0.000)	0.236 (0.068)	0.763 (0.068)
Combined GXR + stimulants (mean SE)				
Normal	0.766 (0.041)	0.231 (0.041)	0.003 (0.001)	0.000 (0.000)
Mild	0.112 (0.014)	0.817 (0.016)	0.070 (0.011)	0.000 (0.000)
Moderate	0.003 (0.001)	0.258 (0.019)	0.737 (0.019)	0.003 (0.001)
Severe	0.000 (0.000)	0.000 (0.000)	0.361 (0.102)	0.550 (0.102)

*CGI-S* Clinical Global Impression-Severity, *GXR* guanfacine extended release, *SE* standard error

### Cost data

All analyses were performed from a Canadian Ministry of Health (MoH; Ontario) and a societal perspective. All costs are expressed in 2013 Canadian dollars. Costs estimated before 2013 were adjusted to April 2013 levels based on the health component of the Canadian Consumer Price Index. No costs were discounted because the time horizon of this economic evaluation did not exceed 1 year.

The costs included in the analysis from a MoH perspective were those associated with medication and health care resources used in the management of ADHD. The unit cost of GXR for each available dose was taken from the Quebec’s Medication List, while the daily cost of each long-acting stimulant was based on daily dose and number of pills according to Canadian data from IMS Brogan for children aged 0–12 years [18]. To date, only the province of Quebec has approved GXR for reimbursement. The approval process for reimbursement in other provinces, including Ontario, was pending at the time of the manuscript. The unit cost of each dose of long-acting stimulant was taken from the Ontario Drug Benefit Formulary [19]. The weighted average cost for each stimulant was estimated based on the Canadian market shares for each dose available for the year 2012 provided by IMS Brogan (Table 1).

Costs associated with health care resources used in the management of ADHD were based on a study by Guevara et al. [25]. In this study, resource utilization of specific categories of health care services including primary care visits, mental health visits, pharmacy fills, emergency department visits and hospitalizations were estimated for children with and without ADHD. Unit costs from Canadian sources were applied to the additional resource use estimates associated with ADHD (resource use for children with ADHD – resource use for children without ADHD) to obtain the cost associated with each category of health care service [26, 27]. The mean cost of a script in Canada was obtained from IMS Health Canada and was a weighted average of the mean cost per script of brand and generic products [28]. The same number of non-ADHD pharmacy fills was applied to all patients.

Medical costs derived from the study by Guevara et al. were allocated according to disease severity. More specifically, the annual medical costs for patients in the “normal” health state were assumed to be the same as the median medical costs for non-ADHD patients ($CA245). The cost of the “mild” subgroup has been estimated as follows: the annual cost of the 50^th^ patients (out of 100) with ADHD is $CA322 (median cost) and the minimal cost for an ADHD patient is $CA245 (cost without ADHD). Assuming a linear distribution, the annual cost of the 3.52^th^ patient (the initial proportion of patients in the “mild” state was 3.52 %) was estimated at $CA250. Thus, the average cost for the “mild” subgroup was $CA248 (mean of $CA245 and $CA250). To properly represent the skewedness of the data, the costs incurred by the “severe” patients were assumed to be two times the mean cost estimated from Guevara et al. ($CA738). Therefore, the annual cost in the “severe” group was estimated at $CA1,476. The average annual cost in the “moderate” subgroup was then calculated using the cost estimates of the “mild” and “severe” states and to retrieve the original mean cost estimated from Guevara et al. according to the initial distribution of patients. Therefore the mean annual cost incurred by the “moderate” state was estimated at $CA709 [16, 25].

The costs of productivity loss associated with ADHD were added from a societal perspective. These costs were estimated by a literature review of published productivity loss data. As no Canadian study was retrieved, productivity loss-related data expressed in number of hours or days were preferred. Hakkart van Roijen et al. reported that the mean number of days absent from work per year was over 17 days for parents of ADHD children in the Netherlands [29]. This number of days was applied on a weekly basis, based on the average Canadian national hourly wage in January 2013 ($CA23.50) [30]. In order to estimate productivity losses associated with ADHD disease severity, the same ratios calculated for medical costs were applied to the weekly cost associated with productivity losses.

### Utility

Utility values associated with the model’s health states were taken from a study by Lloyd et al. [31]. In this study, a survey was carried out among 100 members of the general population in the United Kingdom in order to estimate utility values associated with ADHD-related health states. Utility values estimated from the time trade-off method were used (Table 1). The different ADHD-related health states found in this study were based on the CGI-S and were defined similarly to the ones used in the present model, with the exception that the severe state excluded CGI-S 7 (“*among the most extremely ill patients*”), because no data were available for this CGI-S score in Lloyd et al. [31].

### Adverse events

AEs included in the product monograph that impacted at least 5 % of all treatment arms were considered. In the base-case analysis, AEs were assumed to result in a utility decrement lasting for 4 weeks. Disutilities associated with AEs were assigned after conducting a literature review of published utility scores associated with ADHD treatment-related AEs. The incidences of AEs for both treatment arms and disutilities associated with AEs were published previously [17].

### Incremental cost-effectiveness analyses

Effectiveness outcomes included average quality-adjusted life years (QALYs), response rate, number needed to treat (NNT) and patient-weeks with a response. The incremental QALYs were calculated as the difference in the average QALYs over the time horizon between the two comparators. Similarly, the incremental response rate was calculated as the difference in response rate for GXR with stimulants at week 8 and response rate for stimulants alone at week 8. The NNT was calculated as 1/(response rate for GXR – response rate for stimulants alone). The incremental patient-week with a response was calculated as the difference in the cumulative fraction of patients in either a mild or moderate state over the 52-week period for the two comparators. The incremental cost-effectiveness ratios (ICERs) were calculated by dividing the difference in total costs of the GXR + stimulant arm and the stimulant monotherapy arm by the difference in effectiveness outcomes between both treatment arms. The incremental cost per patient-week with a response was taken by dividing the incremental cost by the incremental patient-weeks with a response.

### Source of data

Data used to perform this economic evaluation were taken from different sources. Data freely available comprised cost of medications taken from provincial (Quebec and Ontario) drug formularies, cost associated with medical management of ADHD and other medical costs taken from published literature as well as utilities estimates. Data that were not freely available comprised patient-level data from the pivotal study to estimate the transition probabilities and data on market shares and cost per script. These data were obtained from Shire and IMS Brogan respectively.

### Sensitivity analyses

To confirm the robustness of the base-case results, several one-way sensitivity analyses were performed by varying a single variable individually within lower and upper bounds of all key parameters including: transition probabilities, costs, utilities, duration of AEs, stimulant choice, and initial state distribution. More specifically, sensitivity analysis on transition probabilities were performed using the observed transitions between the health states during the first 8 weeks and assuming that health states were stabilized without further transitions in the second stage of the model (i.e., week 9 to week 52).

In the base-case model, ordered logit models were used to estimate the transition probabilities. A last observation carried forward (LOCF) technique was used in sensitivity analysis to obtain transition probabilities. The LOCF technique was applied when data were missing from a particular timeslot, but existed before that slot. This technique creates efficacy records for missing visits by carrying data from previous visits forward. One exception was that observations from the baseline timeslot were never carried forward into the treatment phase timeslots.

In most Canadian provinces, long-acting stimulants are listed on the Drug Benefit Formulary [32]. However, their coverage is often provided under specific conditions and patients have to meet several criteria, which may greatly limit the access to long-acting stimulants. To take this situation into account, a sensitivity analysis comparing GXR adjunctive therapy to short/intermediate-acting stimulants with placebo plus short/intermediate-acting stimulants was performed by varying only the stimulant drug costs.

In addition, a probabilistic sensitivity analysis (PSA) was performed to assess the overall impact of uncertainty associated with study parameters. Simultaneous variations in all key parameters were performed using Monte Carlo simulation. A total of 10,000 Monte Carlo simulations were performed using appropriate distributions (beta distribution bounded by 0 and 1 for transition probabilities and utilities, triangular distribution for costs, and uniform distribution for the duration of all AEs). Results of the PSA were presented as cost-effectiveness acceptability curves and the probability of being cost-effective at a threshold of $50,000/QALY was estimated, which is a commonly cited threshold by Canadian agencies for health technology assessment [33].

## Results

### Base-case analysis

Over a 1-year time horizon, GXR as an adjunctive therapy to long-acting stimulants was associated with an average of 0.655 QALYs, compared to an average of 0.627 QALY with long-acting stimulants as monotherapy, for a gain of 0.028 QALY (Table 3). From a MoH perspective, GXR as an adjunctive therapy to long-acting stimulants and long-acting stimulants as monotherapy were associated with total costs of $CA1,617 and $CA949, respectively (difference of $CA668), which resulted in an ICER of $CA23,720/QALY. From a societal perspective, GXR as an adjunctive therapy to long-acting stimulants and long-acting stimulant monotherapy were associated with total costs of $CA3,915 and $CA3,582, respectively (difference of $CA334), which resulted in an ICER of $CA11,845/QALY. In addition, the incremental response rate at week 8 was 13.5 %, which led to a NNT at week 8 of 7.41. The incremental cost per patient-week with response was estimated at $CA102/responder and at $CA51/responder from a MoH and a societal perspective, respectively.

**Table 3:** Cost-effectiveness results – base-case analysis

	Long-acting stimulant monotherapy	GXR + long-acting stimulant	Incremental^a^
Average QALYs	0.627	0.655	0.028
Patient-weeks with response	12.46	19.03	6.57
Drug costs, $CA	337	1,072	735
Medical costs, $CA	612	545	−67
Productivity losses, $CA	2,633	2,299	−334
Total cost, $CA MoH perspective	949	1,617	668
Total cost, $CA Societal perspective	3,582	3,915	334
Incremental cost/QALY, $CA MoH perspective			**$CA23,720/ QALY**
Incremental cost/QALY, $CA Societal perspective			**$CA11,845/QALY**
Incremental cost/patient-week with response, $CA MoH perspective			**$CA102/responder**
Incremental cost/patient-week with response, $CA Societal perspective			**$CA51/responder**

^a^May not sum to total because of rounding

*GXR* guanfacine extended release, *MoH* Ministry of Health, *QALY* quality-adjusted life year

### Sensitivity analysis

**Fig. 2:**
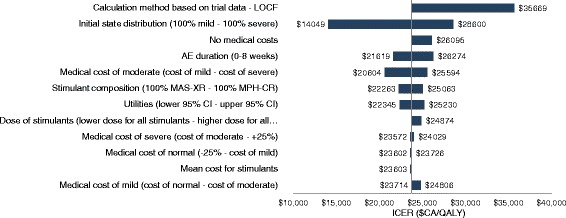
Results of one-way sensitivity analysis. Results of one-way sensitivity analysis are presented in a Tornado diagram from a Ministry of Health perspective. Lower and upper bounds for considered for the sensibility analysis are indicated on the y-axis for each parameter. The base-case icremental cost-effectiveness ratio is $CA23,720/QALY. AE: adverse event; CI: confidence interval; ICER: incremental cost-effectiveness ratio; LOCF: last observation carried forward; MAS-XR: amphetamine mixed salts; MPH-CR: methylphenidate HCl controlled release; QALY: quality-adjusted life year

**Fig. 3:**
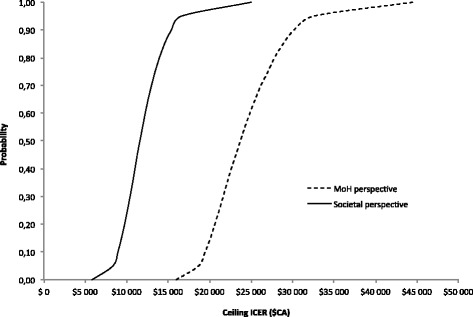
Results of probabilistic sensitivity analysis. Results of probabilistic sensitivity analysis are presented in cost-acceptability curves. Dashed line is from a MoH perspective while solid line is from a societal perspective. The commonly cited threshold in Canada is $CA50,000/QALY. ICER: incremental cost-effectiveness ratio; MoH: Ministry of Health; QALY: quality-adjusted life year

According to the one-way sensitivity analysis results, the ICER of GXR as an adjunctive therapy to long-acting stimulants compared to long-acting stimulant monotherapy varied between $CA14,049/QALY and $CA35,669/QALY from a MoH perspective. The parameters with the greatest impact on base-case ICERs from the MoH perspective were (i) the calculation of transition probabilities based on trial data for the first 8 weeks and then LOCF for the remainder of the study period and (ii) the initial health state distribution assuming 100 % of patients started in the severe state (Fig. 2). From a societal perspective, results of the one-way sensitivity analyses showed that GXR as an adjunctive therapy to a long-acting stimulant was a dominant alternative compared to long-acting stimulant monotherapy when (i) 100 % of patients were assumed to start in the severe state and (ii) productivity losses of the moderate health state were assumed to be equivalent to that of the severe health state. In a sensitivity analysis where patients were maintained on treatment and could transition between heath states during the weeks 9-52 period the ICERs increased to $47,909 and $36,034 from a MoH and a societal perspective respectively. According to a willingness to pay threshold of $CA50,000/QALY, GXR as an adjunctive therapy to long-acting stimulants was a cost-effective alternative over long-acting stimulant as monotherapy in 100.0 % of the Monte Carlo simulations, from both MoH and societal perspectives (Fig. 3).

## Discussion

This economic evaluation indicates that, compared to long-acting stimulants as monotherapy, GXR as an adjunctive therapy to long-acting stimulants is a cost-effective alternative among children with ADHD with a suboptimal response to stimulants. Results of comprehensive sensitivity analyses confirm the robustness of the base-case results.

This is the first Canadian economic evaluation of GXR as an adjunctive therapy in the treatment of ADHD. The results of a previous CEA performed from a US third-party payer perspective suggested that GXR as an adjunctive therapy to long-acting stimulants in the treatment of children and adolescents with ADHD who had a suboptimal response to stimulants was cost-effective according to a willingness to pay threshold of $US50,000/QALY, with an ICER of $US31,660/QALY [17]. The present economic evaluation is a Canadian adaptation of the US study published by Sikirica et al. [17]. In the US study, the estimated ICER was higher than that calculated for the Canadian adaptation ($CA23,720/QALY from a MoH perspective). These differences could be explained by the higher treatment cost of GXR in the US study and the higher weekly medical costs. Nevertheless, the findings of both studies were similar and thus strengthen the conclusion that GXR as an adjunctive therapy to long-acting stimulants is a cost-effective alternative compared to long-acting stimulants as monotherapy.

This economic evaluation has several strengths. First, the model provides results in terms of cost per responder, which gives further evidence on the cost-effectiveness of GXR as an adjunctive therapy to a long-acting stimulant in the treatment of children with ADHD. Moreover, the analysis accounted for AEs associated with treatments as well as productivity losses associated with disease severity, thus allowing a broader perspective and perhaps a more representative assessment of all the impacts of the disease and intervention. Lastly, the comparison of GXR as an adjunctive therapy to short/intermediate-acting stimulants with short/intermediate-acting stimulants alone allowed considering the fact that a significant proportion of patients with ADHD in Canada may receive short/intermediate-acting stimulants because of the reimbursement criteria and specific conditions required by Canadian provinces for the coverage of long-acting stimulants [32].

However, this economic evaluation also has some limitations. The difference in number of QALYs between the two treatments is small, but the difference in number of patient-weeks with response also indicates a gain in efficacy with the adjunctive therapy. As for any model-based analysis, in the absence of data, assumptions were made that may increase the uncertainty of the results. First, although the two-stage model was consistent with an approach used in a health technology assessment conducted by the National Institute for Health and Care Excellence, it was assumed that patients discontinued their treatment if they transitioned into a moderate or a severe state during the second stage of the simulation (weeks 9–52) [22]. Although this may not always be observed in clinical practice, the latter assumption may be realistic because most patients are considered to achieve optimal dosing within the first 8 weeks of treatment and because a significant proportion of patients discontinue their medication by the end of 1 year anyway [34, 35]. Furthermore, it was assumed that patients who discontinued treatment did not receive subsequent therapy. Although this may not be representative of the real-world clinical setting, there was insufficient clinical evidence or consensus in treatment guidelines on the management of patients who were non-responsive to adjunctive therapy. Another limitation of the current study involves the availability of data regarding cost parameters. As medical costs and productivity losses associated with disease severity have not been reported, some assumptions were made based on existing studies in order to estimate these parameters. Although costs associated with productivity losses usually represent the main cost component specific to a societal perspective, other costs, such as “out-of-pocket” expenses can be also considered with a societal perspective. In this study, only the addition of costs associated with productivity losses was considered for the societal perspective. Moreover, cost of long-acting stimulants was based on daily dose and number of pills according to Canadian data for children aged 0–12 years, and therefore, specific data for patients aged 6–12 years, which corresponded to the target population in the study, were not available. However, patients aged less than 6 years would be on much lower doses in general. As lower doses used by very young children may contribute to lower the mean daily cost of comparators, this would potentially be a conservative assumption. Similarly, because the OCCI Costing Analysis Tool divided age into three categories (0–17, 18–69 and 70+ years), some cost parameters associated with health care resource utilization did not precisely reflect costs specific for the target age of the population in the present study. Despite these limitations, findings of this cost-utility analysis are robust according to the base-case and confirmed by the robust one-way and probabilistic sensitivity analyses. Lastly, one other limitation should be mentioned. As the Wilens et al. study was designed and powered collectively for only those long-acting stimulants described previously, future research may need to study whether there are differences in efficacy among the individual long-acting medications combinations when GXR was added to them, or those medications that are on the market within Canada but were not in the US clinical trial.

## Conclusion

This economic evaluation suggests that, from both a societal and a Canadian health care system perspective, GXR as an adjunctive therapy to long-acting stimulants is a cost-effective strategy compared to long-acting stimulant monotherapy in the treatment of children with ADHD.

## Abbreviations

ADHD: Attention-deficit/hyperactivity disorder
ADHD-RS-IV: Attention-deficit/1hyperactivity disorder rating scale version IV
AE: Adverse event
CEA: Cost-effectiveness analysis
CGI-S: Clinical global impressions of severity of illness
GXR: Guanfacine extended release
ICER: Incremental cost-effectiveness ratio
LDX: Lisdexamfetamine dimesylate
LOCF: Last observation carried forward
MAS-XR: Amphetamine mixed salts
MoH: Ministry of Health
MPH-CR: Methylphenidate HCl controlled release
NNT: Number needed to treat
OROS-MPH: Methylphenidate HCl extended release
PSA: Probabilistic sensitivity analysis
QALY: Quality-adjusted life year
